# Supplementary material for: Aspirin and Primary Cancer Risk Reduction in Ischemic Cardiac or Cerebrovascular Disease Survivors: A Nationwide Population-Based Propensity-Matched Cohort Study
Source: Cancers (Basel). 2022 Dec 23;15(1):97. doi: 10.3390/cancers15010097 (PMC9817941; doi:10.3390/cancers15010097)
Supplement: Supplementary file 1 [file cancers-15-00097-s001.zip › cancers-1938273-supplementary.pdf]

Table S1. Demographic and baseline clinical characteristics of patients in the two propensity-matched study cohorts who had aspirin use or matched index date within 90 days after ICCD

Characteristics	Aspirin user (*n* = 1141)		Aspirin nonuser (*n* = 1141)		Standardized mean difference
	n	%	*n*	%	
**Sex**					
Female	530	46.5	527	46.2	<0.001
Male	611	53.6	614	53.8	<0.001
**Age (years)**					
20–39	17	1.49	50	4.38	0.210
40–59	204	17.9	241	21.1	0.060
60–79	620	54.3	489	42.9	0.260
≥ 80	300	26.3	361	31.6	0.160
**Comorbidities**					
Hypertension	825	72.3	829	72.7	0.010
Diabetes mellitus	405	35.5	417	36.6	0.060
Hyperlipidemia	332	29.3	335	29.6	0.010
Atrial fibrillation	102	9.01	109	9.63	0.020
Alcohol-related illness	34	2.98	47	4.12	0.020
Mild liver disease	373	32.7	348	30.5	0.040
Moderate or severe liver disease	31	2.72	29	2.54	0.010
Hepatitis B	42	3.68	40	3.51	0.040
Hepatitis C	26	2.28	30	2.63	0.050
Rheumatic disease	81	7.10	73	6.40	<0.001
Myocardial infarction	141	12.4	150	13.2	0.010
Congestive heart failure	281	24.6	284	24.9	0.040
Chronic pulmonary disease	270	23.7	258	22.6	0.002
Peptic ulcer disease	558	48.9	538	47.2	0.060
Gastrointestinal bleeding	270	23.7	258	22.6	0.020
Renal disease	121	10.6	131	11.5	0.010
**Prior drug use**					
Anti-hypertensive agents	912	79.9	912	79.9	0.049
Drugs for cardiac therapy	625	54.8	641	56.2	0.010
Peripheral vasodilators and vasoprotectives	376	33.0	381	33.4	0.010
HMG-CoA reductase inhibitors	96	8.41	86	7.54	0.030
Other lipid-modifying agents	27	2.37	22	1.93	0.060
Hypoglycemia agents	358	31.6	378	33.4	0.040

Coumadin and heparin	187	16.5	186	16.4	0.002
Other antithrombotic agents	355	31.4	339	30.0	0.030
Proton pump inhibitors	297	26.2	271	23.9	0.050
H2-receptor antagonists	416	36.8	395	34.9	0.040
Antacids	936	82.7	927	81.9	0.020
Estrogens and progestogens	31	2.74	28	2.47	0.020
Non-steroidal anti-inflammatory drugs	784	69.3	809	71.5	0.050

HMG-CoA, 3-hydroxy-3-methyl-glutaryl-coenzyme A; H2-receptor, histamine type-2 receptor.

Prior drug use was defined as medications that were prescribed within 6 months prior to the index date.

Standardized mean difference ≤ 0.2 indicates a negligible difference between the two study cohorts.

Table S2. Incidence rate and risk of developing primary cancer in ICCD survivors who had aspirin use or matched index date within 90 days after ICCD, stratified by various types of cancer.

**Cancer (ICD-9-CM)**	**Aspirin user**		**Aspirin nonuser**		**Aspirin user vs. Aspirin nonuser**	
	**Event**	**IR**	**Event**	**IR**	**Crude HR (95 % CI)**	**Adjusted HR (95 % CI)**
**All (140–208)**	49	7.83	73	43.6	0.26 (0.18–0.37)***	0.23 (0.15–0.34)***
Head and neck (140–149)	0	0.00	5	2.99	-	-
Esophagus (150)	1	0.16	2	1.20	-	-
Stomach (151)	4	0.64	7	4.18	0.23 (0.06–0.80)*	0.17 (0.04–0.74)*
Colon (153-154)	12	1.92	21	12.6	0.21 (0.10–0.43)***	0.20 (0.09–0.43)***
Hepatoma (155)	7	1.12	11	6.57	0.20 (0.08–0.54)**	0.28 (0.09–0.83)*
Pancreas (157)	1	0.16	1	0.60	0.34 (0.02–5.70)	-
Lung (162)	12	1.92	13	7.77	0.39 (0.17–0.87)*	0.33 (0.14–0.78)*
Breast (174, 175)	2	0.32	2	1.20	0.34 (0.05–2.45)	-
Ovary (183)	0	0.00	0	0.00	-	-
Prostate (185)	10	1.60	11	6.57	0.35 (0.14–0.83)*	0.22 (0.08–0.61)**

ICCD, ischemic cardiac or cerebrovascular disease; IR, incidence rates (per 1,000 person-years); HR, hazard ratio; CI, confidence interval.

In the Cox proportional hazard model, adjusted HR was obtained after adjustment for age, sex, baseline comorbidities, and prior drug use.

*$p < 0.05$, **$p < 0.01$, ***$p < 0.001$.
